# Development and validation of a prediction nomogram for depressive symptoms in gout patients

**DOI:** 10.3389/fpubh.2024.1356814

**Published:** 2024-07-19

**Authors:** Xinyi Hao, Aiping Wang

**Affiliations:** ^1^Public Service Department, The First Hospital of China Medical University, Shenyang, China; ^2^Nursing Department, Peking Union Medical College Hospital, Peking, China

**Keywords:** immunotherapy, gout, depression, nomogram, prediction model

## Abstract

**Objective:**

The objective of the study was to explore the risk factors for depressive symptoms in patients with gout and to construct and validate a nomogram model.

**Methods:**

From October 2022 to July 2023, a total of 469 gout patients from a Class iii Grade A hospital in Northeast China were selected as the research objects by the convenience sampling method. The General Information Questionnaire, Self-Rating Depression Scale, Gout Knowledge Questionnaire, Self-Efficacy Scale for Managing Chronic Disease (SEMCD), and Social Support Rating Scale were used to conduct the survey. Univariate and multivariate logistic regression analyses were used to establish a depression risk prediction model and construct a nomogram. The bootstrap method was used to verify the performance of the model.

**Results:**

The detection rate of depressive symptoms in gout patients was 25.16%. Binary logistic regression analysis showed that male, the number of tophi, acute attack period, lack of knowledge about gout, the number of attacks in the past year, and the duration of the last attack were independent risk factors for post-gout depression. Female, interictal period, chronic arthritis period, knowledge of gout, and social support were protective factors for post-gout depression (*p* < 0.05). The calibration (χ^2^ = 11.348, *p* = 0.183, *p* > 0.05) and discrimination (AUC = 0.858, 95%CI: 0.818–0.897) of the nomogram model for depressive symptoms in gout patients were good.

**Conclusion:**

The prevalence of depressive symptoms in gout patients is high, and it is affected by gender, current disease stage, number of tophi, gout knowledge level, the number of attacks in the past year, and the last attack days. The nomogram model is scientific and practical for predicting the occurrence of depressive symptoms in gout patients.

## 1 Introduction

Gout is a chronic ailment triggered by a disruption in the body’s purine metabolism (1). This metabolic imbalance leads to an accumulation of monosodium uric acid crystals, either in joints or non-joint areas, subsequently inducing joint inflammation or causing damage to surrounding tissues. Its clinical manifestations are diverse, including hyperuricemia, acute gouty arthritis, tophus formation, gouty nephropathy, joint deformity, and dysfunction, among others (2). This condition has systemic effects, often impacting multiple organs and potentially causing widespread damage. When gout flares up, patients typically experience acute and intense pain in their peripheral joints, accompanied by redness, heat, and lasting discomfort that can persist for hours. As the condition progresses, the deposition of monosodium uric acid crystals increases, elevating the likelihood and severity of tophus occurrence. This can lead to joint damage, deformation, and even disability, significantly affecting the patient’s mobility and overall quality of life (3).

In recent years, alterations in lifestyle have contributed to a yearly increase in gout cases in China, with a noticeable trend toward younger patients (4). Gout, currently considered the second most prevalent metabolic disease after diabetes, poses a significant threat to individuals’ physical and mental wellbeing. Economic growth, changes in dietary habits, and evolving lifestyles have led to a gradual global increase in gout prevalence. Currently, gout rates vary from 0.1 to 6.8% across different countries, with a reported prevalence of 1.1% in China (5). Recent statistics reveal that the number of individuals with high uric acid hematologic disorders in our country has escalated to 170 million, with over 14.66 million gout patients (6). Studies indicate that gout affects more men than women and is showing a trend toward younger age groups, with the disease burden also on the rise (7, 8).

Research has revealed that gout has a recurrence rate of up to 63% (9). Repeated flare-ups and the excruciating pain they bring significantly impact patients’ quality of life and healthcare consumption. These flare-ups can cause considerable mental health deterioration, leading to anxiety, depression, or other psychological issues (10). Consequently, this can result in decreased compliance with gout treatments (11). Early identification and targeted interventions can alleviate the psychological stress on gout patients, thereby enhancing their adherence to treatment and improving their overall quality of life (12). However, current research on depression among gout patients remains limited to status surveys, and there is a lack of a reliable tool to identify depressive symptoms specifically tailored for this patient population. Therefore, this study aimed to explore the factors associated with depressive symptoms in gout patients and develop a risk prediction model based on a nomogram. This model will provide valuable evidence for the early identification and prevention of depressive symptoms in gout patients.

## 2 Materials and methods

### 2.1 Study population

Using the convenient sampling method, gout patients who were treated in the Department of Rheumatology and Immunology of a Class iii Grade A hospital in Shenyang, Liaoning Province, from November 2022 to July 2023 were selected as the research objects. The inclusion criteria were as follows: ① meeting the diagnostic criteria of the 2015 American College of Rheumatology (ACR) guidelines (13); ② age ≥ 18 years old and disease duration ≥1 year; ③ communicating normally and cooperating to complete the questionnaire; and ④ willing to participate in the study and sign the informed consent form. The exclusion criteria were as follows: ① previous psychiatric history or history of cognitive and mental disorders and ② patients had a definite diagnosis of depression before gout diagnosis. This study was approved by the medical ethics committee of the hospital where the study was conducted.

### 2.2 Data collection

The following survey instruments for data collection were used: ① General Information Questionnaire: The questionnaire was self-designed by the research team based on the results of our previous research and a literature search, which included socio-demographic factors such as gender, age, body mass index (BMI), education level, marital status, occupation, medical insurance, family *per capita* monthly income, and residence. Family history, course of disease, number of tophi, cumulative number of joints, current disease stage, number of attacks in the past year, duration of the last attack, number of chronic diseases, and other disease-related data. ② Self-rating Depression Scale (SDS) was used to evaluate the depression of gout patients. The scale contained 20 items; each item was scored on a Likert four-point scale, ranging from “no or little time” to “most or all time” (14). The original crude score was multiplied by 1.25 to calculate the standard score. When the total score of the standard score is ≥53, the patient has depression. ③ Gout Knowledge Questionnaire (GKQ) consisted of 10 questions (15). Each item was correctly answered with one point, the total score was 0–10, and a score ≥ 7 was considered to be gout-related knowledge. The Flesch–Kincaid readability score of the questionnaire was 4.7, and the Flesch readability was 81.4%. ④ Self-Efficacy Scale for Managing Chronic Disease (SEMCD) (16): The scale was designed by Lorig et al. from Stanford University in the study of self-management behavior of patients with chronic diseases. It contained a total of 6 items, which reflected the self-efficacy of patients with chronic diseases in many aspects, including symptom management, role function, emotional control, and communication with doctors. Each item was scored from 1 to 10, with 1 indicating no confidence and 10 indicating complete confidence. The average score of the six items reflected the level of self-efficacy, and the higher the score, the higher the self-efficacy. The scale is easy to use and has been widely used. Cronbach’s α coefficient was 0.87, and the test–retest reliability was 0.91. ⑤ Social Support Rating Scale (SSRS): The revised version of the social support scale developed by Xiao Shuiyuan in 1990 was adopted, including three dimensions and 10 items (17). The three dimensions were: objective support (the actual support received by patients), subjective support (the emotional support or support that can be experienced by patients), and social support utilization (the active utilization of various social supports by patients). In the Social Support Rating Scale, the correlation coefficient between the three subscales and the total scale ranged from 0.724 to 0.835, and Cronbach’s α coefficient was 0.780.

### 2.3 Statistical analysis

SPSS26.0 was used for statistical analysis, including the chi-square test, independent sample *t*-test, and logistic regression analysis. Statistical significance was set at *p* < 0.05. All statistical tests were two-tailed. RStudio software was used to construct the risk prediction model. Nomogram was used for model visualization. The discrimination ability of the nomogram was measured using the Harrell concordance index (C-index). The receiver operating characteristic (ROC) curve was used to evaluate the diagnostic efficacy of the model. Consistency between actual and nomogram-predicted generalization probabilities was assessed using calibration curves (1,000 resampling bootstraps) in the internal verification.

## 3 Results

### 3.1 Univariate analysis of depressive symptoms in gout patients

A total of 469 gout patients were included in the care; 118 cases (25.16%) had depression, whereas 351 cases (74.84%) did not have depression. There were statistically significant differences in the incidence of depressive symptoms in gout patients with different genders, marital status, number of tophus, disease stage, level of gout-related knowledge, number of attacks in the past year, duration of the last attack, number of chronic diseases, pain degree, number of joints involved, self-efficacy, and social support (*p* < 0.05). There was no significant difference in the incidence of depressive symptoms among gout patients with different ages, body mass index, education levels, family monthly income *per capita*, working status, medical insurance status, permanent residence, family history, and family support (*p* > 0.05), as shown in Table 1.

**Table 1:** Comparison of the incidence of depressive symptoms in gout patients with different characteristics.

	Number of cases	Non-depressed (*n* = 351)	Depressed (*n* = 118)	*X* ^2^	*p*
Gender					
Male	400	314	86	19.341	< 0.001
Female	69	37	32		
Age				4.402	0.111
18–43	262	205	57		
44–59	95	59	36		
60~	51	26	25		
Body mass index (BMI)				0.682	0.878
Too low	21	16	5		
Normal	288	218	70		
Overweight	113	84	29		
Obesity	47	33	14		
Marital status				7.871	0.049
Unmarried	91	69	22		
Married	350	267	83		
Divorce	15	9	6		
Widowed	13	6	7		
Level of education				3.667	0.300
Junior high school and below	127	103	24		
High school or technical secondary school	114	82	32		
Undergraduate or junior college	146	106	40		
Graduate student and above	82	60	22		
Household *per capita* monthly income				3.301	0.347
<3,000	62	46	16		
3,000~	146	108	38		
5,000 ~	146	104	42		
>7,500	115	93	22		
Working status				0.962	0.327
Unemployed	177	128	49		
Employed	292	223	69		
Medical insurance				0.017	0.895
Have not	58	43	15		
Have	411	308	103		
Residence				0.117	0.732
Rural areas	141	107	34		
Cities	328	244	84		
Family history				0.527	0.468
Have	104	75	29		
Have not	365	276	89		
Numbers of tophus				56.399	< 0.001
No tophus	286	248	38		
Single tophi	131	77	54		
Multiple tophi	52	26	26		
Disease stages				23.845	< 0.001
Acute attack period	135	83	52		
Interval period	279	231	48		
Chronic arthritis stage	55	37	18		
Knowledge of gout				11.634	0.001
Do not know	327	230	97		
Know	142	121	21		
Number of attacks in the last year	469	2.93 ± 3.013	4.70 ± 2.655	5.687	< 0.001
Duration days	469	4.40 ± 2.733	5.69 ± 2.846	4.362	< 0.001
Number of comorbidities	469	0.80 ± 0.931	1.07 ± 1.002	2.617	0.009
Level of pain	469	6.07 ± 1.827	6.69 ± 2.032	2.97	0.003
The number of joints involved	469	2.26 ± 1.817	3.60 ± 2.173	6.026	< 0.001
Self-efficacy	469	6.75 ± 2.112	5.32 ± 1.982	6.424	< 0.001
Social support	469	36.57 ± 7.886	31.10 ± 7.753	6.547	< 0.001

### 3.2 Binary logistic regression analysis of depressive symptoms in gout patients

Taking gout patients with depressive symptoms as the dependent variable (assignment: 0 = no, 1 = yes) and the statistically significant factors in the univariate analysis as the independent variables (assignment of independent variables is shown in Table 2), binary logistic regression analysis was performed, *α*_in_ = 0.05, *α*_out_ = 0.10. Regression analysis showed that male, the number of tophi, acute attack period, lack of knowledge about gout, the number of attacks in the past year, and the duration of the last attack were independent risk factors for post-gout depression. Female, intermittent period, chronic arthritis period, gout knowledge, and social support were the protective factors for post-gout depression (*p* < 0.05) (Table 3).

**Table 2:** Assignment of independent variables.

Independent variables	Assignment of value
Gender	Male = 1, Female = 2
Marital status	Unmarried = 1, Married = 2, Divorced = 3, Widowed = 4
Number of tophi	No tophi = 1, Single tophi = 2, Multiple tophi = 3
The present stage of the disease	Acute attack period = 1, Interval period = 2, Chronic arthritis stage = 3
Knowledge of gout	Do not know = 1, Know = 2
Number of attacks in the last year	Measured values
Days of duration	Measured value
Number of comorbidities	Measured values
Level of pain	Measured value
Number of joints involved	Measured values
Self-efficacy	Measured values
Social support	Measured values

**Table 3:** Binary logistic regression analysis of depressive symptoms in gout patients.

Variables of interest	B	SE	Wald *X*^2^	OR (95%CI)	*p*
Constant	1.394	0.999	1.949		
Gender	1.398	0.352	15.772	4.048 (2.03 ~ 8.071)	< 0.001
The number of tophi	0.736	0.206	12.782	2.087 (1.394 ~ 3.123)	< 0.001
Stage of illness (acute attack period)			10.765		0.005
Stage of illness (interval period)	0.939	0.297	10.007	0.391 (0.219 ~ 0.7)	0.002
Stage of illness (chronic arthritis stage)	0.992	0.475	4.362	0.371 (0.146 ~ 0.941)	0.037
Knowledge of gout	0.706	0.322	4.814	0.494 (0.263 ~ 0.927)	0.028
The number of attacks in the past year	0.144	0.049	8.482	1.155 (1.048 ~ 1.272)	0.004
The number of days the most recent episode lasted	0.159	0.045	12.375	1.173 (1.073 ~ 1.281)	< 0.001
Social support	0.087	0.019	20.171	0.917 (0.883 ~ 0.952)	< 0.001

### 3.3 Construction of a risk prediction model for depressive symptoms in gout patients

**Figure 1:**
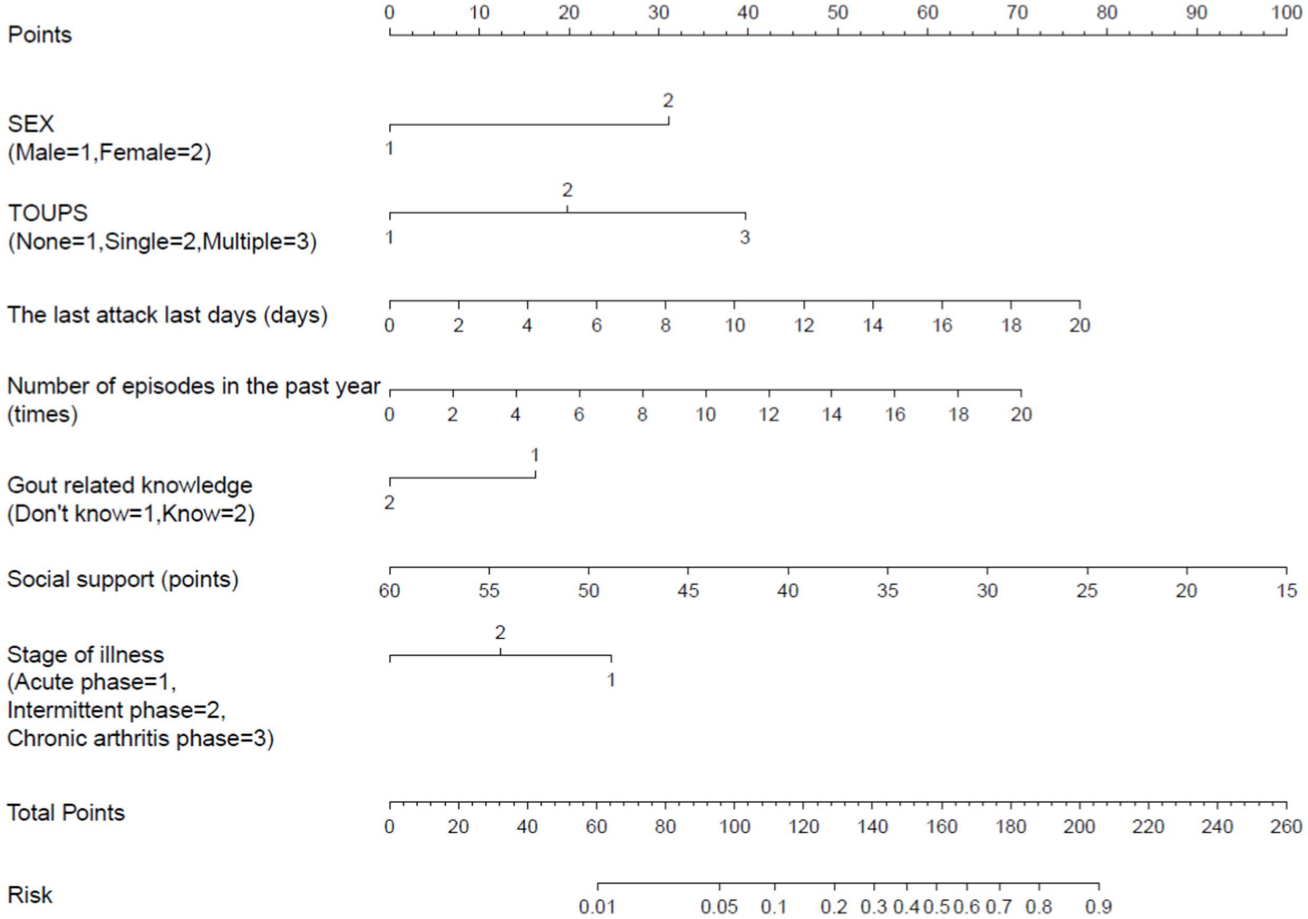
Gout patients with depressive symptoms nomogram of the risk prediction model.

**Figure 2:**
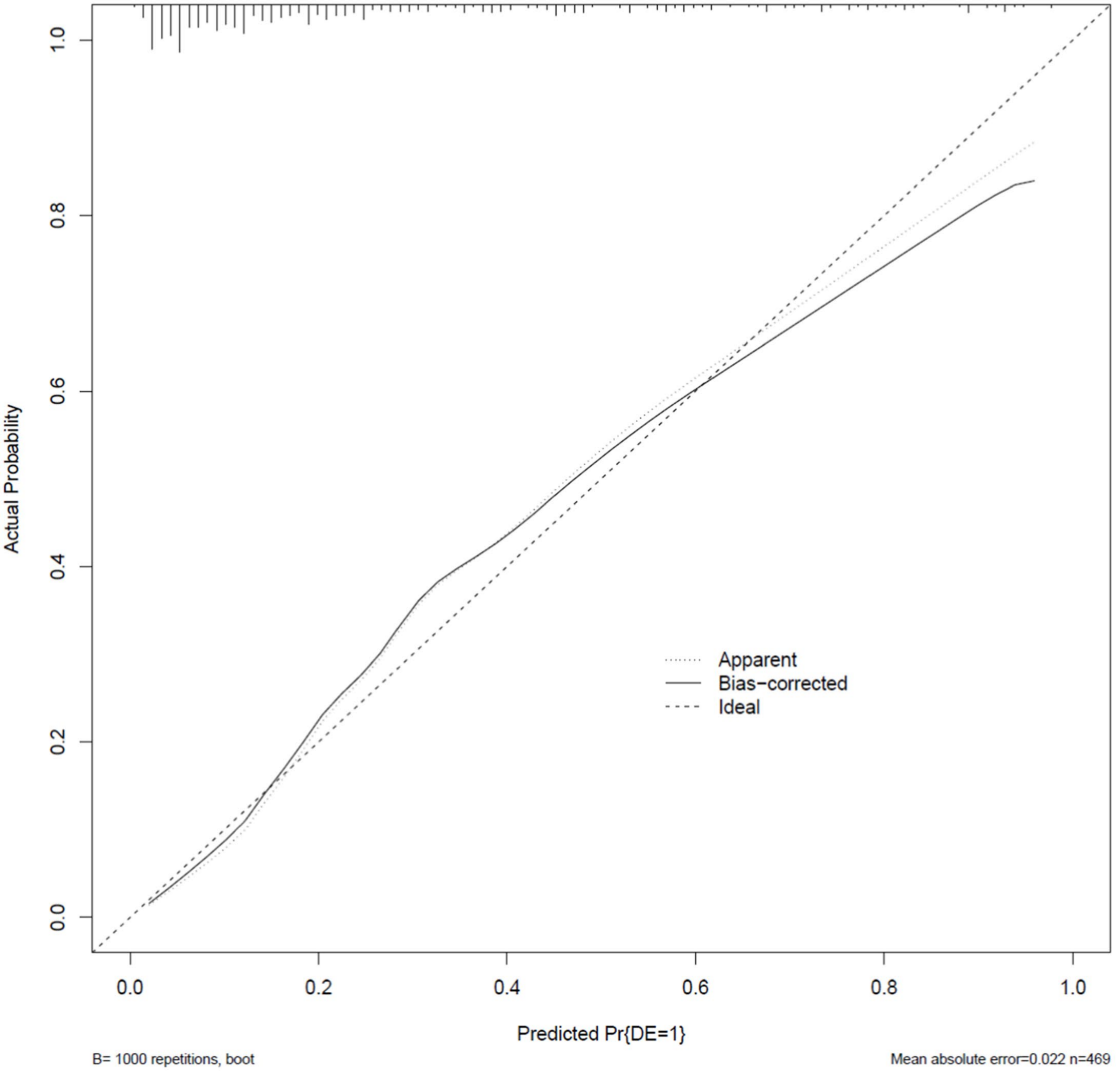
Gout patients with depression risk prediction nomogram classification calibration curve of the model.

**Figure 3:**
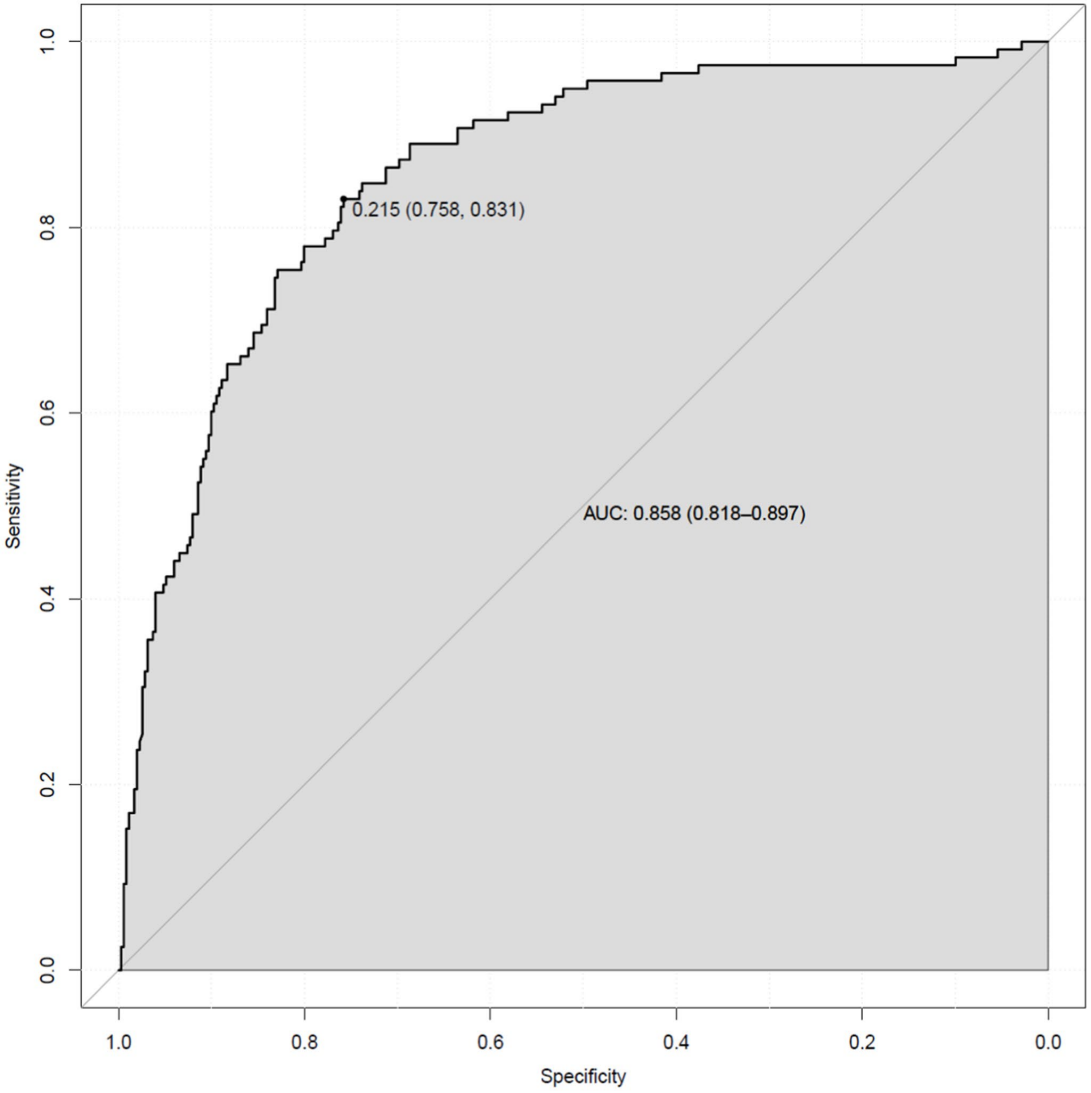
Gout patients with depression risk prediction nomogram model of the ROC curve.

**Figure 4:**
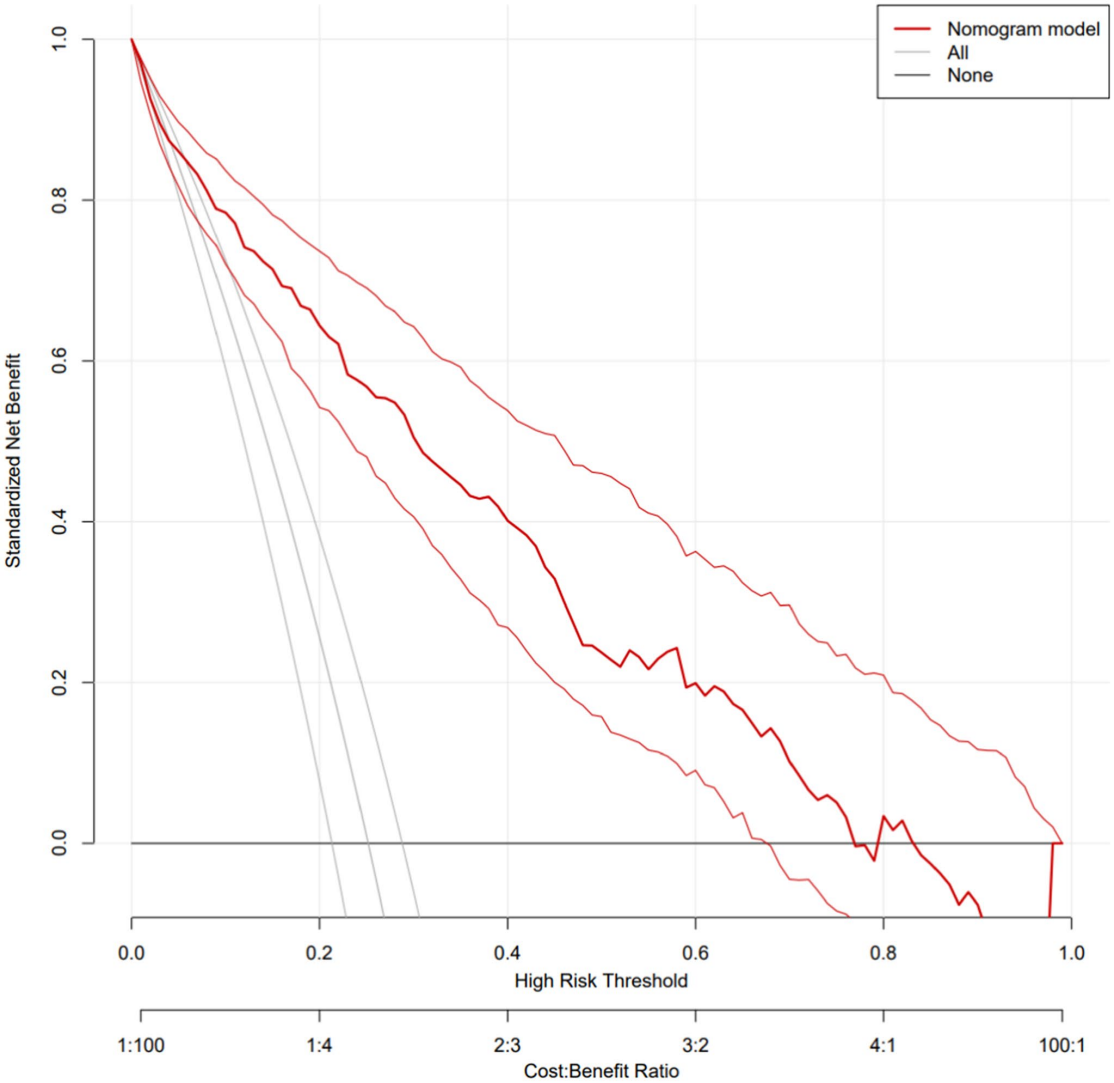
Gout patients with depression risk prediction nomogram DCA curves of the model.

Based on the independent factors entered into the regression model (gender of gout patients, number of tophi stones, current disease stage, number of attacks in the past year, duration of the last attack, gout knowledge level, and social support), a risk prediction model for depression in caring for gout patients was constructed, and a nomogram was constructed, as shown in Figure 1. As shown in the first line of the “points” as a standard score, with the “gender” to illustrate, when evaluating patients with sex for women, the corresponding score is about 31.5; in this way, it is concluded that the patient with the score of seven variables, in the “total points” found on the location of the total score, should draw a line straight down. The number at the intersection of this line and “risk” is the risk probability of depression in this gout patient. The high coincidence degree between the line of data points and the diagonal oblique line indicated that the prediction model had a good calibration, as shown in Figure 2. The area under the ROC curve (AUC) was 0.858 (95% CI, 0.818–0.897), indicating that the model had good discrimination, as shown in Figure 3. The red curve drawn by the prediction model, which is higher than the two extreme lines, indicates that the clinical practical value of the prediction model is good and patients can benefit from it, as shown in Figure 4. The bootstrap method was used for internal validation of the model, and the area under the ROC curve (AUC) was 0.860 (0.857–0.862), indicating that the model had good discrimination. The Hosmer–Lemeshow test results showed that χ^2^ = 11.348, *p* = 0.183 (*p* > 0.05), suggesting that the prediction model had good consistency.

## 4 Discussion

The psychological status of patients with gout is poor due to the characteristics of prolonged gout, repeated attacks, and severe pain during attacks. A meta-analysis showed that gout is positively correlated with depression (18). The results of this study showed that the detection rate of depressive symptoms in gout patients was 25.16%, which was similar to the results of Ding Yayi’s study (19) and lower than the results of Chen Qiuzhi’s study (20). On the one hand, gout patients have severe pain due to repeated attacks of the disease, which can lead to joint deformation or tophus in severe cases, seriously affecting the body image. On the other hand, it is necessary to control the intake of purine for many years to manage the disease, which puts forward more stringent requirements for the management of the daily life of gout patients, leading to depressive symptoms. Therefore, attention should be paid to the psychological status of gout patients during health education or nursing intervention, and psychological AIDS should be used to intervene in time for patients with depression tendency.

### 4.1 Analysis of influencing factors of depressive symptoms in gout patients

The results showed that female, the number of tophi, acute attack period, the number of attacks in the past year, the duration of the last attack, and the lack of knowledge about gout were independent risk factors for post-gout depression. Male, intermittent period, chronic arthritis period, awareness of gout knowledge, and social support are protective factors for post-gout depression. The results of this study showed that the incidence of depressive symptoms in female patients with gout was 4.048 times that in male patients, which was consistent with the results of Chen’s study (20). Women are more likely to have anxiety or depression symptoms than men, possibly due to personality characteristics and hormone secretion. Hormone fluctuations may play a role in the increased risk of depression in women, especially during the menopausal transition period (21). The severity of gout can be reflected by the number of tophi, the current disease stage, the number of attacks in the past year, and the duration of the last attack. This study shows that patients with multiple tophi have a higher risk of depressive symptoms than those with a single tophi or those without tophi, and studies have shown that the number and dysfunction of tophi are the main contributors to depression in gout patients (22). Compared to the gout interictal stage and chronic arthritis stage, patients in the acute attack stage of ventilation have higher depressive symptoms, which may be closely related to the severe joint pain caused by a gout attack. This study also showed that the higher the number of gout attacks in a year and the duration of gout attacks, the higher the risk of depressive symptoms in gout patients, which was consistent with Fu’s study (12). Recurrent acute attacks of gout not only bring physical pain to patients but also affect their normal life, work, and social function. It will have a negative impact on patients from both physical and psychological aspects and seriously damage their quality of life (22).

Related studies have shown that a higher level of social support has a strong positive effect on the mental health of gout patients (23), which is consistent with the results of this study. Social support can be various forms of recognition, support, and help provided by family, friends, neighbors, colleagues, etc. (24). When social support plays a positive role, it will have a positive effect on the physical and mental health of gout patients. Gout patients with a higher level of social support can invest more energy in self-management and psychological regulation and have more confidence to deal with various challenges in the process of disease management so that they can face the disease more actively with a high level of self-management efficacy and thus reduce the occurrence of depression and other negative emotions due to disease recurrence. Therefore, when managing gout patients, medical staff should also provide relevant training for their family members, use effective intervention methods to provide long-term psychological counseling for gout patients with depression tendencies, eliminate their subjective bad feelings, and relieve their physical and mental pressure.

Nomogram, a plane graph with graduated line segments based on regression results, is used to assess the scientific and practical validity of the depression risk prediction model for gout patients. Its essence is the visualization of regression equations (25). In this study, seven variables entered into the regression equation model were integrated to construct a nomogram model. The results showed that the nomogram had good discrimination and calibration [AUC = 0.860 (0.857–0.862)], and the H-L deviation test results showed that χ^2^ = 11.348, *p* = 0.183 (*p* > 0.05). Based on the nomogram, the individual measured variable values were perpendicular to the first line to find the corresponding points. After adding the corresponding scores of each variable value, a point was found at the total points. The risk of depressive symptoms in gout patients was found to be perpendicular to the last line. The nomogram-based risk prediction model for depression in gout patients is scientific and practical and can provide personalized and high-accuracy risk estimation for post-gout depression in gout patients. At the same time, because the risk factors are visualized by the nomogram model, medical staff can estimate the risk of depressive symptoms in gout patients according to the model so as to find the tendency for depression early and then carry out effective interventions to prevent the occurrence of depression.

## 5 Limitations

The present study has several limitations. First, the AUC is larger for the validation cohort than the development cohort. This is probably due to the small sample size and the high variability (as a result of the small sample size in the validation cohort). Meanwhile, this also reflected that the prediction model was not overfitted. Second, the related factors included in the model are limited, and we should consider continuing to include more influencing factors of post-gout depression to further supplement the results (such as the patient’s diet and medication, and the amount of vitamin D and uric acid in the blood). Finally, this study only included outpatients with gout in one tertiary hospital in northeast China, so the selection of the sample size was limited. Despite internal validation demonstrating good discrimination and calibration of the nomogram model, we did not perform external validation. Hence, external validation using other ethnic populations and centers with a larger-scale sample size is warranted to further improve the prediction performance of the model.

## 6 Conclusion

The detection rate of depressive symptoms in gout patients is high. This study has established a risk prediction model for depression in this population, which is conducive to early identification of depression in this population, and the model has good test efficiency.

## Data availability statement

The raw data supporting the conclusions of this article will be made available by the authors, without undue reservation.

## Ethics statement

The studies involving humans were approved by the First Hospital affiliated to China Medical University Ethics Committee. The studies were conducted in accordance with the local legislation and institutional requirements. The participants provided their written informed consent to participate in this study.

## Author contributions

XH: Writing – original draft. AW: Writing – review & editing.
